# A Multivariate Meta-Analysis for Optimizing Cell Counts When Using the Mechanical Processing of Lipoaspirate for Regenerative Applications

**DOI:** 10.51894/001c.143940

**Published:** 2025-09-30

**Authors:** Gershon Zinger, Nia Kepes

**Affiliations:** 1 Hand Unit, Department of Orthopedic Surgery Shaare Zedek Medical Center, Faculty of Medicine, Hebrew University of Jerusalem, Jerusalem 91120, Israel; 2 Department of Neuroscience Michigan State University Lyman Briggs College, East Lansing, MI 48824, USA

## 25

Lipoaspirate has become the preferred source for regenerative cells. The mechanical processing of lipoaspirate has advantages over enzymatic processing but has a lower yield of regenerative cells. A review of the literature shows different techniques of extraction, but the ideal method or combination has not been determined. Methods: A comprehensive literature search was focused on the mechanical processing of lipoaspirate, without the use of enzymes. Data from the articles were integrated by utilizing a multivariate meta-analysis approach and used to create a statistical-based predictive model for a combination of multiple variables. Results: Starting with 10,000 titles, 159 articles were reviewed, and 6 met the criteria for inclusion and exclusion. The six studies included data on 117 patients. Sixteen factors were analyzed and six were identified as significant. The predictive profilers indicated that the optimal combination to maximize the cell yield was: a centrifuge force of 2000× g, a centrifuge time of 10 min, a cannula diameter of 2 mm, and an intra-syringe number of passes of 30. The optimal patient factors were a higher BMI and younger age. Conclusions: The novelty of the method used here was in combining data across different studies to understand the effect of the individual factors and in the optimization of their combination for mechanical lipoaspirate processing.

